# Anti-hepatofibrosis effect of *Allium senescens* in activated hepatic stellate cells and thioacetamide-induced fibrosis rat model

**DOI:** 10.1080/13880209.2018.1529801

**Published:** 2018-12-04

**Authors:** Gwang-Mo Shin, Sushruta Koppula, Yun-Jin Chae, Hyun-Su Kim, Jae-Dong Lee, Myong-Ki Kim, MinDong Song

**Affiliations:** a Department of Applied Life Science, Graduate School of Konkuk University, Chungju-si, Chungbuk, Republic of Korea;; b Department of Biotechnology, College of Biomedical and Health Sciences, Konkuk University, Chungju-si, Chungbuk, Republic of Korea;; c Daesowon Food, Chungju-si, Chungbuk, Republic of Korea;; d Department of Internal Medicine, School of Medicine, Konkuk University, Chungju, Chungbuk, Republic of Korea;; e Department of Food Science and Engineering, Seowon University, Cheongju, Chingbuk, Republic of Korea

**Keywords:** Liver fibrosis, thioacetamide, extracellular matrix, apoptosis, hydroxyproline

## Abstract

**Context:***Allium senescens* Linn. (Liliaceae) (ASL) has been traditionally used in Korea and other Asian countries for improving digestive and liver functions.

**Objective:** The anti-hepatofibrosis effect of ASL ethanol extract in cellular and experimental fibrosis rat model was investigated.

**Materials and methods:***In vitro* cell viability, cell cycle and apoptosis in hepatic stellate cells (HSCs) were studied using MTT assay, flow cytometry and Annexin V-FITC/PI staining. Thioacetamide (TAA; 200 mg/kg, *i.p.*)-induced liver fibrosis model using Sprague Dawley rats (*n* = 10) was developed *in vivo* by injecting TAA twice per week for 13 weeks. ASL (25 and 100 mg/kg) and silymarin (50 mg/kg) were administered through oral gavage 2 times per week from 7th to 13th week. Specific fibrotic-related biomarkers such as aspartate transaminase (AST), alanine transaminase (ALT), glutathione and hydroxyproline levels in serum were analyzed by spectrophotometer using commercial kits. Morphological, histopathological and fibrotic-related gene expression such as TGF-β, Col1α1 and α-SMA in liver tissues was estimated by hematoxylin and eosin staining, Picrosirius red stain and quantitative real-time polymerase chain reaction, respectively.

**Results:** ASL (0.1 mg/mL) and silymarin (0.05 mg/mL) treatment induced apoptosis (4.06% and 8.67%) in activated HSC-T6 cells, compared with control group (3.7%). The altered morphology in activated primary HSCs was also restored by ASL (0.1 mg/mL) treatment. Further, ASL (100 and 25 mg/kg) ameliorated the TAA-induced altered fibrotic-related biomarkers, histopathological changes and fibrotic-related gene expression significantly (*p <* 0.05 ∼ *p <* 0.001).

**Conclusions:** ASL can potentially be developed as a therapeutic agent in the treatment of hepatic fibrosis.

## Introduction

Liver fibrosis, a common consequence of chronic liver injuries is characterized by excessive extracellular matrix (ECM) deposition (Xu et al. 2014). Historically, liver fibrosis was known as an irreversible process due to the destruction of liver tissue and the accumulation of collagen. It is initiated by a variety of insults prominently viral infections such as hepatitis B and C virus, alcohol abuse and nonalcoholic fatty liver disease leading to the death of hepatocytes (Lurie et al. 2015). However, clinical reports have suggested that advanced liver fibrosis is potentially reversible (Soyer et al. 1976). It is well known that hepatic stellate cells (HSCs) play one of the important roles in the liver fibrosis. HSCs are usually quiescent cells, but in response to liver injury they undergo an activation process in which they become highly proliferative and synthesize a fibrotic matrix rich in type I collagen (Reeves and Friedman 2002) Indeed, activated HSCs are key players increasing hepatic vascular tone and also synthesizing ECM components (Pinzani and Gentilini 1999).

Accumulation of ECM proteins distorts the hepatic architecture by forming a fibrous scar, and the subsequent development of nodules of regenerating hepatocytes defines liver fibrosis (Bataller and Brenner 2005). Therefore, it is important to induce the apoptosis of HSCs or prevent the secretion of the ECM by HSCs (Lee et al. 2014). Several studies have been documented on the prevention or treatment of liver fibrosis through natural herbs (Fakurazi et al. 2008; Kim et al. 2017; Koppula et al. 2017; Yum et al. 2017). *Allium senescens* Linn. (Liliaceae) is a perennial aromatic herb found abundantly in Asian and European countries. The whole plant including the stems, roots and leaves are added in diet to cleanse circulatory system, control cholesterol levels and aid in proper digestion (Fern 1996). As a food ingredient and children’s snack, *A. senescens* is used as a cracker with retrograded rice powder (Ha et al. 2014). *Allium senescens* extract was also used in traditional medicine to treat liver disease with detoxification effects and in restoring liver function (Kim 1998). However, no scientific evidence exists on the beneficial effects of *A. senescens* on hepatofibrosis. Therefore, in the present study, we investigated the anti-hepatofibrotic effects of *A. senescens* extract in an *in vitro* HSC-T6 cells and *in vivo* thioacetamide (TAA)-induced hepatic fibrosis rat models.

## Materials and methods

### Reagents and chemicals

TAA, silymarin, hydroxyproline, *p*-dimethylaminobenzaldehyde, 1,1,3,3-tetraethoexypropane, chloramine-T, 5,5-dithiobis-2-nitrobenzoic acid (DTNB), and glutathione (GSH), standards of chlorogenic acid, *p*-coumaric acid, and rutin were purchased from Sigma (St. Louis, MO). Dulbecco’s modified Eagle’s medium (DMEM) and fetal bovine serum (FBS) were acquired from Invitrogen (Carlsbad, CA). Perchloric acid was obtained from GFS Chemical Co. (Columbus, OH). GOT-GPT assay kit was purchased from Asan Pharmaceutical (Hwaseong-si, Korea). Annexin V-FITC and propidium iodide (PI) Apoptosis Detection Kit I was acquired from BD Biosciences (San Jose, CA). All other reagents used in this study were of the highest grade available commercially.

### Plant material and extraction

The plant material of *A. senescens* collected during March–April, 2017 was purchased from Sanchewon Co., Ulleung, South Korea. The material was authenticated by Prof. Jong-Bo Kim a Taxonomist, at Konkuk University, South Korea, based on its microscopic and macroscopic characteristics. A voucher specimen (ASL-KU2017) was kept in our department herbarium for future reference. For extraction, shade dried whole plant of *A. senescens* (100 g) was ground to a fine powder and extracted with 1 L ethanol (99.9%) using Soxhlet’s technique for 3 days. The extract was then concentrated in vacuum under reduced pressure and lyophilized. The final yield of the lyophilized *A. senescens* extract named ASL, was 5.27% (*w*/*w*) and stored at 4 °C. The lyophilized powder of ASL was dissolved in 10% dimethyl sulfoxide (DMSO) and then filtered through a 0.22 μM syringe filter and stored as stock until use for each experiment. The final concentration of DMSO used for the study was not more than 0.1%.

### Animals and cell cultures

Fifty healthy male specific-pathogen-free Sprague Dawley (SD) rats (6 weeks old, 190–210 g) were purchased from a commercial animal breeder (Orient Bio, Gyeonggi-do, Korea). Animals were housed in conventional cages under control conditions of temperature (23 ± 3 °C), relative humidity (50 ± 20%) and 12 h light/dark cycle with free access to a standard diet and sterile water. The study was approved by the Committee of Laboratory Animals according to institutional guidelines of Konkuk University, Korea (IACUC No. KU17163).

For cell cultures, Chang liver cell line was purchased from ATCC (Manassas, VA). Chang liver cell line was used as a normal human cell line derived from normal liver tissue. The cells were cultured in DMEM (GIBCO, Carlsbad, CA) supplemented with 10% FBS (GIBCO), 1% antibiotic–antimycotic (Invitrogen) in a humidified atmosphere of 5% CO_2_ at 37 °C. An immortalized rat HSC lines (HSC-T6) generously received by Prof. Chang-Gue Son (Korean Hospital of Daejeon University, Korea) were cultured in DMEM supplemented with 5% FBS, 1% antibiotic–antimycotic in a humidified atmosphere of 5% CO_2_ at 37 °C. Quiescent HSC cells were activated by serum starvation after FBS supplementation.

For primary HSCs culture, HSCs were isolated from 7-week-old male SD rats by in situ pronase, collagenase perfusion and single-step Histogenz gradient as reported previously (Hendriks et al. 1985; Nair and Seo 1993). Isolated HSCs were cultured in low glucose DMEM (GIBCO) containing 10% FBS (GIBCO) and 1% antibiotic–antimycotic (Invitrogen) on uncoated plastic maintained in a humidified atmosphere of 5% CO_2_ at 37 °C and these activated HSCs were used in the experiments. Growth medium was changed on a daily basis for 7 days.

### Cell viability assay

Cell viability assays were measured using 3-(4,5-dimethylthiazol-2yl)-2,5-diphenyl-2*H*-tetrazolium bromide (MTT) method. In a 96-well plate, HSC-T6 (6 × 10^5^ cells/well) were cultivated in DMEM medium supplemented as described previously (Vogel et al. 2000). The effect of ASL on cell viabilities was evaluated by treatment with various concentrations (0.01, 0.025, 0.1, 0.25 and 0.5 mg/mL) for 24 h at 37 °C in an atmosphere of 5% CO_2_ and 95% humidity. The cells were then incubated with 0.5 mg/mL MTT (Sigma) for 3 h, and the reaction was interrupted by addition of DMSO (JUNSEI, Tokyo, Japan). An ELISA reader was used to obtain the results at 540 nm. The viabilities of the control cells were used as the control values at 100%.

### Cell cycle analysis

HSC-T6 cells (15 × 10^5^ cells/well) were cultured in DMEM containing 10% FBS (GIBCO) and 1% antibiotic–antimycotic (GIBCO) maintained in a humidified atmosphere of 5% CO_2_ at 37 °C. Growth medium was changed on a daily basis for 7 days. For cell cycle analysis, sample materials of ASL (0.1 and 0.025 mg/mL) were evaluated for 24 h at 37 °C in an atmosphere of 5% CO_2_ and 95% humidity. After treatment for 24 h, cells were washed with PBS twice, suspended in 1 mL cold PI solution (50 μg/mL PI and 100 μg/mL RNase A). Next, the cells were incubated on ice for 30 min in the dark and then analyzed with a flow cytometer (FACSCalibur, BD Biosciences).

### Apoptosis analysis

Apoptosis was determined by Annexin V-FITC and PI (FITC Annexin V Apoptosis Detection Kit I, BD Biosciences) and detected according to manufacturer’s instructions. Data analysis was performed with CellQuest software (Beckton Dickinson), which allowed assessing of specific population only. Individualization by gates was according to size (forward scatter (FSC)), granularity (side scatter (SSC)) and fluorescent (FL) parameters. Both early apoptotic (Annexin V^+^ and PI^−^) and late apoptotic (Annexin V^+^ and PI^+^) cells were included in cell death determinations.

### Quantitative real-time polymerase chain reaction (qRT-PCR)

The primers used in the study are shown in Table 1. Total RNA was extracted from liver tissue samples and HSC-T6 cells using TRIzol reagent (QIAGEN, Carlsbad, CA). cDNA was synthesized from total RNA (2 μg) in a 20 μL reaction using a high-capacity cDNA reverse transcription kit (Applied Biosystems, Foster City, CA). For analysis of data, the gene expression levels were compared with those of β-actin as a reference gene.

**Table 1.** The primers used in the study to evaluate Fibrotic related gene expressions.

qRT-PCR	Primer sequences	
α-SMA	Forward	5′-AACACGGCATCATCACCAACT-3′
	Reverse	5′-TTTCTCCCGGTTGGCCTTA-3′
Collagen type 1 α 1	Forward	5′-CCCAGCGGTGGTTATGACTT-3′
	Reverse	5′-GCTGCGGATGTTCTCAATCTG-3′
TGF-β1	Forward	5′-AGGAGACGGAATACAGGGCTTT-3′
	Reverse	5′-AGCAGGAAGGGTCGGTTCAT-3′
β-actin	Forward	5′-CTAAGGCCAACCGTGAAAAGAT-3′
	Reverse	5′-GACCAGAGGCATACAGGGACAA-3′

α-SMA: Alpha-smooth muscle actin, Col1α1: Collagen type-1α1, TGF-β1: Transforming growth factor β-1.

### TAA-induced rat model of acute liver injury and experiment design

After 1 week of acclimation, rats were divided randomly into five groups (*n* = 10). Control group injected with normal saline intra-peritoneally (*i.p*), TAA group, ASL 25 (TAA with 25 mg/kg ASL), ASL 100 (TAA with 100 mg/kg ASL), and positive control silymarin group (TAA with 50 mg/kg silymarin). To induce liver fibrosis, TAA (200 mg/kg) was injected (*i.p*) twice a week for 13 weeks to four groups except control group. ASL (25 or 100 mg/kg), silymarin (50 mg/kg) or distilled water was given by gastric gavage six times per week from the 7th week to the 13th week. After last treatments, animals fasted for 18 h, and then blood was collected by cardiac puncture under CO_2_ anesthesia. The Liver tissues were removed and stored at −80 °C separately and were used for hydroxyproline, GSH content and fibrosis-related gene expressions determination. A portion of the liver tissue fixed in Bouin’s solution was processed for histo-morphological findings and another small portion of liver tissue was fixed in RNA later solution for gene expression studies.

### Serum biochemical analysis

On the final day of the experiment, blood was collected into heparinized tubes *via* the cardiac puncture under CO_2_ anesthesia. Serum was separated using centrifuge (3000 *g*, 15 min) following blood clotting. The serum levels of aspartate transaminase (AST), and alanine transaminase (ALT) were determined by spectrophotometer using a commercially available GOT-GPT assay kit (Asan Pharmaceutical).

### Determination of total GSH content in liver tissues

We measured the levels of GSH using a spectrophotometer according to Ellman’s method (Ellman 1959). Briefly, a 50 µL sample of homogenate (or GSH standard) was combined with 80 µL of freshly prepared DTNB/NADPH mixture (10 µL 4 mM DTNB and 70 µL 0.3 mM NADPH) in a 96-well plate. Finally, 20 µL (0.06 U) of GSH reductase solution was added to each well and the absorbance was recorded at 412 nm after 5 min. The amount of GSH was expressed as mM of GSH per gram of tissue.

### Determination of hydroxyproline in liver tissues

Hydroxyproline determination was conducted as previously described, with some modifications (Takayama et al. 2003). Briefly, liver tissues were homogenized with dilution buffer. Hydrolysis was performed by adding 1 mL of 6N HCl to 2 mL of liver homogenate in a tightly capped glass tube and then incubating samples overnight at 100 °C. After cooling, the acid hydrolysates were filtered through a 0.45 µm filter paper (Toyo Roshi Kaisha, Tokyo, Japan). Next, 50 µL samples or hydroxyproline standards in 6N HCl were air-dried and dissolved in methanol (50 µL), after which 1.2 mL of 50% isopropanol and 200 µL of chloramine-T solution were added to each sample, which was followed by incubation at room temperature for 10 min. Ehrlich’s solution (1.3 mL) was then added, after which the samples were incubated at 50 °C for 90 min and the optical density of the reaction product was read at 558 nm using a spectrophotometer (Sunrise, Tecan, San Jose, CA). Concentrations were then determined based on comparison with a standard curve constructed using serial dilutions of 0.5 mg/mL hydroxyproline solution.

### Histopathology of liver tissue

Liver tissues were fixed in Bouin’s solution and then embedded in paraffin, after which paraffin sections of 5 µm thickness were stained with hematoxylin and eosin (H&E) and Picrosirius red stain (Chevallier et al. 1994). For identification and analysis of collagen expression, the red-stained areas in the Picrosirius red stained sections were measured collagen expression.

### Fingerprint analysis of ASL using high-performance liquid chromatography (HPLC)

ASL was dissolved in 80% methanol and centrifuged for 5 min at 3000 *g*. The supernatant was used for the determination of analytical sample. The components of ASL were determined with a Prominance LC-20A (Shimadzu, Kyoto, Japan) HPLC system, equipped with a CMA-20A communications bus module, LC 20AD liquid chromatograph, SPD-M20A diode array detector, SIL-20AC auto-sampler, DGU-20A3 degasser and CTO-20AC column oven. HPLC analysis was conducted using a Shim-pack VP-ODS (3 × 75 mm, 2.2 μm, Shimadzu). The flow rate of mobile phase was 0.15 mL/min. The chromatogram was monitored at 320 nm. The injection volume was 10 μL. The column temperature was maintained at 30 °C. The mobile phase consisting of 100% acetonitrile (A) and water containing 0.1% formic acid (B) was run with following gradient programs shown in Table 2. The LC/MS-IT-TOF was operated with a nebulizer gas flow rate of 1.5 mL/min, detection voltage of 1.53 kV, Prove voltage of 450 kV. The mass range (*m*/*z*) was 100–1500 amu.

**Table 2.** Mobile phase condition of HPLC.

Time (min)	A% (acetonitrile)	B% (0.1% formic acid)
0	5	95
5	5	95
20	18	82
40	22	78
60	30	70
90	90	10
100	90	10
100.1	5	95
110	5	95

### Statistical analysis

All the results are expressed as the mean ± standard error of the mean (SEM, *n* = 10). Statistical analysis was carried out using one-way analysis of variance (ANOVA) followed by Student’s *t*-test using Graph Pad Prism software version 4.00 (Graph Pad Software Inc., San Diego, CA). Differences between groups were analyzed by one-way ANOVA. The values of *p* < 0.05 were considered as statistically significant.

## Results

### Effect of ASL on the cell viability and primary HSCs morphology

**Figure 1.**
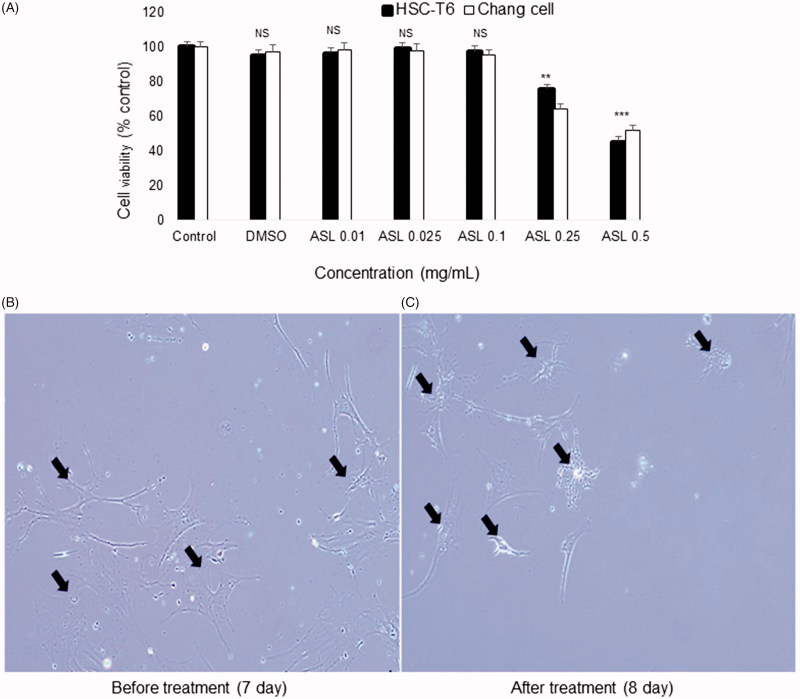
Effect of ASL on cell viabilities in HSC-T6/Chang liver cells and morphological changes in primary HSCs. (A) HSC-T6 and Chang liver cells were incubated with ASL at indicated concentrations for 24 h and the cell viability was determined by MTT assay. (B) Primary HSCs were cultured for 1 week and exposed to the ASL 0.1 mg/mL for 24 h. (C) Pictures were taken before and after 24 h treatment with ASL. Magnification was 100×. Arrows indicate HSCs. The data are expressed as means ± SEM (*n* = 10), using one-way ANOVA followed by Student’s *t*-test. NS: Not significant; **p < 0.01 and ***p < 0.001 compared with control group.

We first assessed the effect of various concentrations (0.01, 0.025, 0.1, 0.25 and 0.5 mg/mL) of ASL on cell viability in HSC-T6 cells and Chang liver cells. As shown in Figure 1(A), ASL at concentrations of up to 0.1 mg/mL did not show any signs of toxicity or changes in cell viability in HSC-T6 cells and Chang liver cells. However, concentrations greater than 0.25 mg/mL induced negative effects on the overall cell viability in both cell lines. Further, the solvent, DMSO (0.1%) used to dissolve the ASL extract did not exhibit any toxicity in HSC-T6 and Chang liver cells. Therefore, in all *in vitro* experiments, we used ASL 0.025 and/or 0.1 mg/mL, as the concentrations were considered effective and nontoxic to study antifibrotic activity *in vitro*.

With respect to the HSCs morphology, untreated activated HSCs showed normal morphology (7th day). Primary activated HSCs treated with ASL at 0.1 mg/mL for 24 h (8th day), showed reduced shrinking collagen fiber morphology and decline in the cell number. In addition, reduced stretched fibers were observed after 24 h when compared to 7 days activated HSCs (Figure 1(B,C)).

### Effect of ASL on the cell cycle analysis and apoptosis in HSC-T6 cells

**Figure 2.**
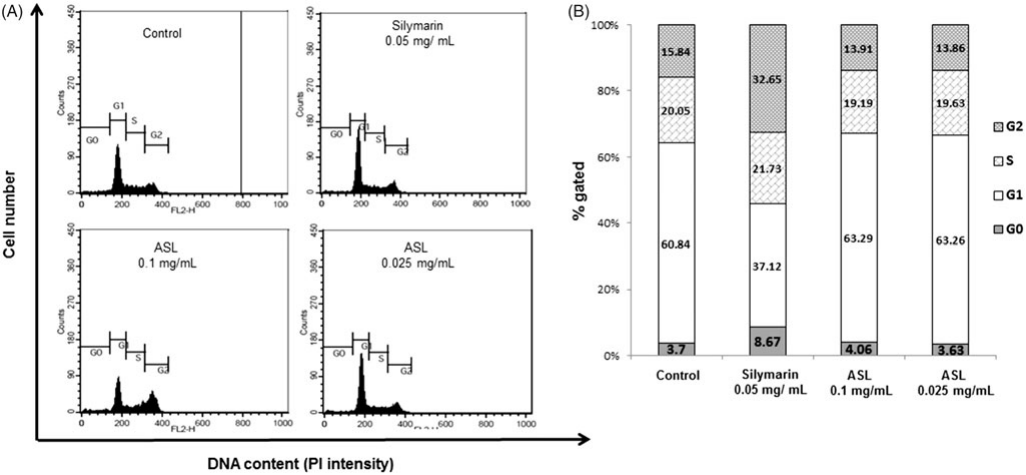
Effect of ASL on the cell cycle in HSC-T6 cells. DNA contents in different phases of the cell cycle were measured using propidium iodide by flow cytometry. The cell cycle distribution and the percentage of the cell cycle distribution were represented by graphs (A) and histogram (B), respectively.

As shown in Figure 2, flow cytometric analysis of ASL (0.25 and 0.1 mg/mL) treated HSC-T6 cells showed a distribution of 3.63 and 4.06% respectively, compared with control cells showing a distribution of 3.7% in the sub-G1(G0) phase. In addition, treatment with silymarin resulted in 8.67% of the cell distribution in the sub-G1 (G0) phase.

**Figure 3.**
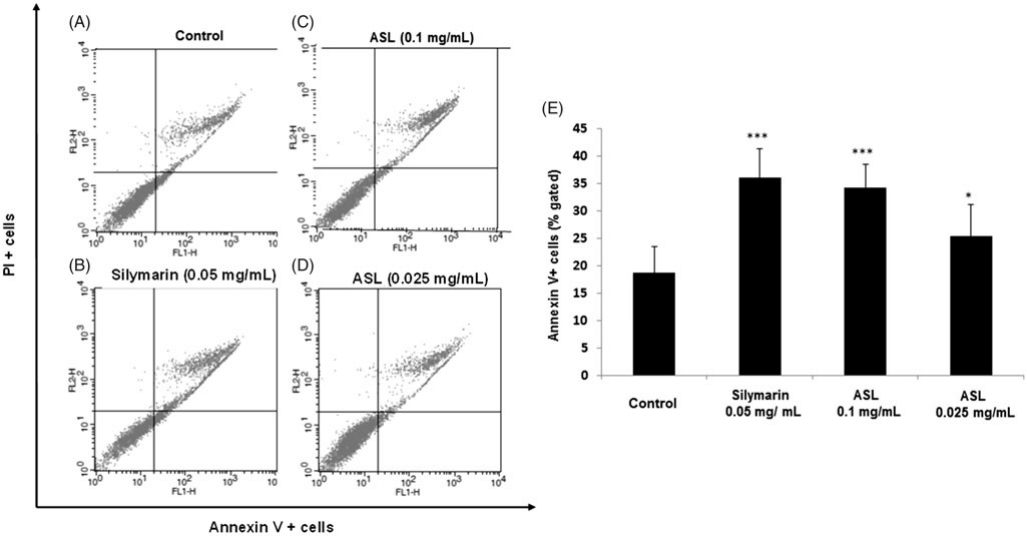
Effect of ASL on apoptosis in HSC-T6 cells. (A) Control cells, (B) flow cytometric data indicate apoptosis in HSC-T6 cells after incubation with Silymarin, (C) ASL 0.1 mg/mL, (D) ASL 0.025 mg/mL for 24 h and (E) data showed the apoptotic (Annexin V^+^ and PI^−^) and late apoptotic (Annexin V^+^ and PI^+^) cells. The data are represented as mean ± SEM (*n* = 10) using one-way ANOVA followed by Student’s *t*-test. *p < 0.05 and ***p < 0.001 compared with control group.

To verify the apoptosis effect of ASL, HSC-T6 cells were treated with silymarin (0.05 mg/mL) and ASL (0.1 and 0.025 mg/mL) for 24 h (Figure 3(A–D)). As shown by the Annexin V-FITC/PI assay, the cells undergoing apoptotic cell death increased significantly from 18.75% ± 4.75 (control group) to 25.42% ± 4.21 (ASL 0.025 mg/mL; *p* < 0.05) and 34.35% ± 5.79% (ASL 0.1 mg/mL; *p* < 0.001). Silymarin treated at 0.05 mg/mL also induced significant (*p* < 0.001) apoptotic effect with 36.21% ± 5.21% (Figure 3(E)). ASL 0.1 mg/mL and silymarin exhibited similar statistical potency when compared with the Annexin V positive control cells (*p* < 0.001). These results indicated that ASL treatment has mild effects including the induction of apoptosis in HSC-T6 cells.

### Effect of ASL on serum biomarkers, total GSH content and hydroxyproline in TAA-induced fibrotic rats

**Figure 4.**
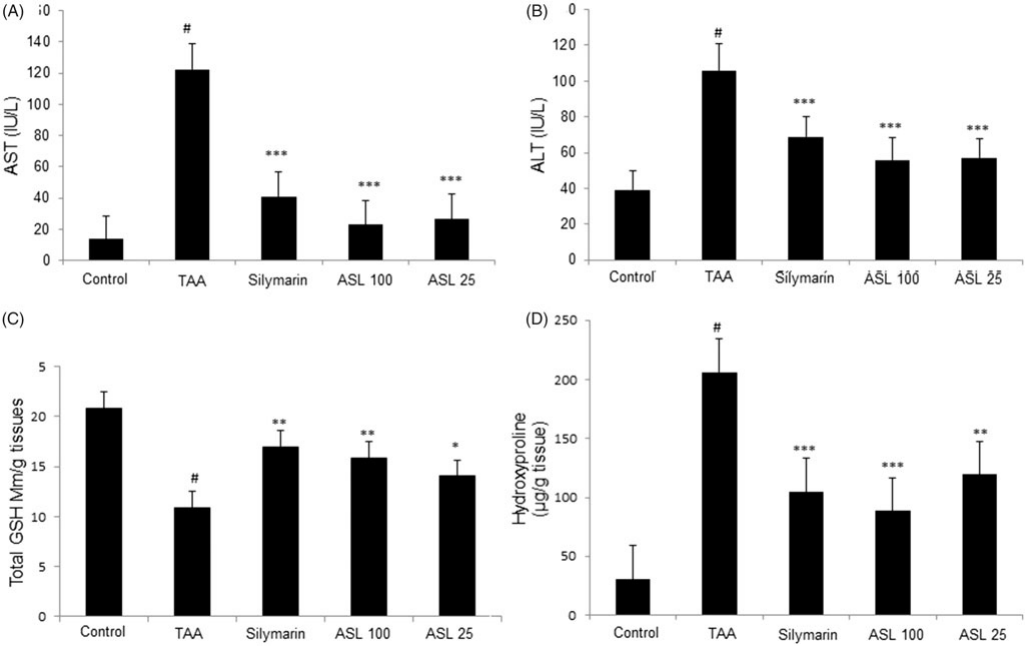
Effect of ASL treatment on liver functions, oxidative stress and fibrosis marker in TAA-induced fibrotic rats. Levels of serum biomarkers ALT (A) and AST (B). Total GSH contents in liver tissue (C) and hydroxyproline levels (D) in TAA-induced liver tissue of rats measured by spectrophotometry. TAA: Thioacetamide-induced liver fibrosis rats; Silymarin: Positive control rats; ASL 100: TAA plus ASL100 mg/kg treated rats; ASL 25: TAA plus ASL 25 mg/kg treated rats. The data are expressed as means ± SEM (*n* = 10) using one-way ANOVA followed by Student’s *t*-test. #*p <* 0.001 as compared with control group, *p < 0.05, **p < 0.01, ***p < 0.001 as compared with TAA-induced group.

TAA-induced group significantly (*p* < 0.001) increased the serum biochemical levels (AST and ALT) when compared with the control group. However, the levels of AST and ALT were significantly (*p* < 0.01 and *p* < 0.001) decreased by ASL (25 and 100 mg/kg) treatment compared with TAA group. Silymarin treatment also showed a positive trend by significantly (*p* < 0.001) attenuating the increased levels of AST and ALT (Figure 4(A,B)). Further, TAA treatment significantly (*p* < 0.001) decreased the GSH contents in the liver tissue when compared with the control group. However, ASL treated at 100 and 25 mg/kg significantly (*p* < 0.05 and *p* < 0.01) attenuated this decrease in GSH contents. The positive control silymarin also significantly (*p* < 0.01) attenuated the decreased GSH content and was similar to ASL 100 treated group (Figure 4(C)). Further, the hydroxyproline content in TAA-induced group was dramatically increased compared to the control group (*p* < 0.001). However, ASL (100 and 25 mg/kg) treatment significantly (*p* < 0.01 and *p* < 0.001) decreased hydroxyproline levels compared with the TAA group. The silymarin treatment also reduced hydroxyproline content significantly (*p* < 0.001) when compared with TAA-induced rats which were similar to ASL 100 treated group (Figure 4(D)). These findings indicate that TAA causes liver fibrosis through decreased ALT and AST serum biomarkers, reactive oxygen species (ROS) damage, and altered hydroxyproline levels which are associated with hepatic fibrosis processes, and that ASL significantly ameliorated these changes.

### Histopathology of liver tissues

**Figure 5.**
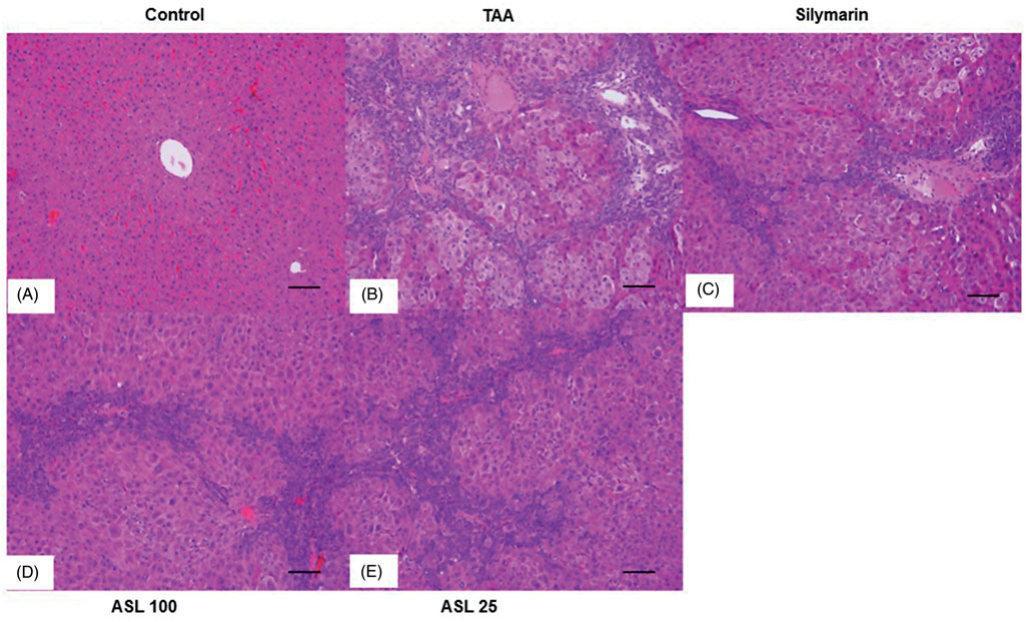
Effect of ASL on TAA-induced morphology of liver tissues by Hematoxylin and Eosin (H&E) staining. Hepatic sections stained with H&E (A–E) (×200). (A) Control, (B) TAA: TAA-induced liver fibrosis rats, (C) silymarin: positive control rats, (D) ASL 100: TAA plus ASL 100 mg/kg treated rats and (E) ASL 25: TAA plus ASL 25 mg/kg treated rats, Scale bar = 200 μM.

**Figure 6.**
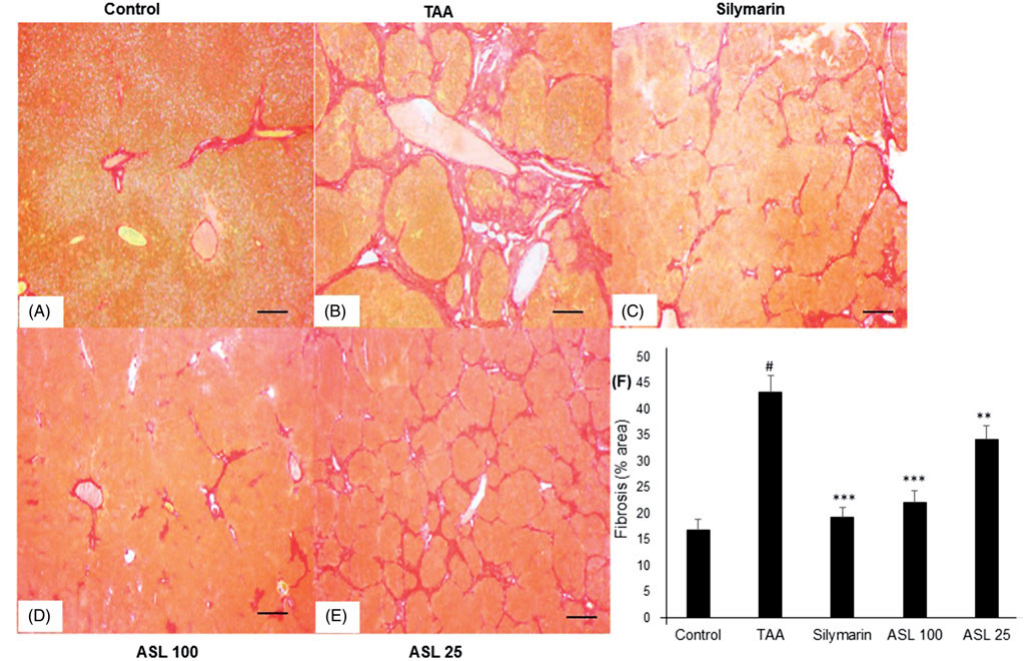
Effect of ASL on TAA-induced fibrosis by Picrosirius red stain of liver tissues. (A) Control group, (B) TAA: TAA-induced liver fibrosis rats, (C) silymarin: positive control rats, (D) ASL 100: TAA plus ASL 100 mg/kg treated rats, (E) ASL 25: TAA plus ASL 25 mg/kg treated rats and (F) percentage of fibrosis area plot. Scale bar = 200 μM. Quantification was done using ImageJ. Values are represented as mean ± SEM (*n* = 10) using one-way ANOVA followed by Student’s *t*-test. #*p <* 0.001 as compared with control group, **p < 0.01, ***p < 0.001 as compared with TAA group. TAA: Thioacetamide.

As shown in Figure 5, the liver tissue section in the control group showed normal state and morphology (Figure 5(A)). TAA-induced group showed severe pathological alterations, with shrunken, solidified and abnormally patterned liver (Figure 5(B)). However, silymarin and ASL (25 and 100 mg/kg) treated groups markedly attenuated the altered liver pattern (Figure 5(C–E)). We further examined the antifibrotic effects of ASL using Picrosirius red staining technique (Figure 6). The liver section treated with TAA led to a fiber optic dysplasia with increased production of collagen and irregular organizational pattern when compared with the control group (Figure 6(A,B)). However, treatment with silymarin and ASL (25 and 100 mg/kg) showed improved morphology (Figure 6(C–E)). Fibrosis percentage area revealed significant damage in TAA treated group compared with the control group (*p* < 0.001). However, ASL at both concentrations ameliorated these changes significantly (*p* < 0.05 for ASL 25 and *p* < 0.001 for ASL 100 group). Silymarin exhibited significant (*p* < 0.001) attenuating affect similar to ASL 100 treated group when compared with TAA alone treated group (Figure 6(F)). Overall, the data indicated that ASL ameliorates liver fibrosis.

### Effect of ASL on fibrosis-related gene expression in TAA-induced liver tissues

**Figure 7.**
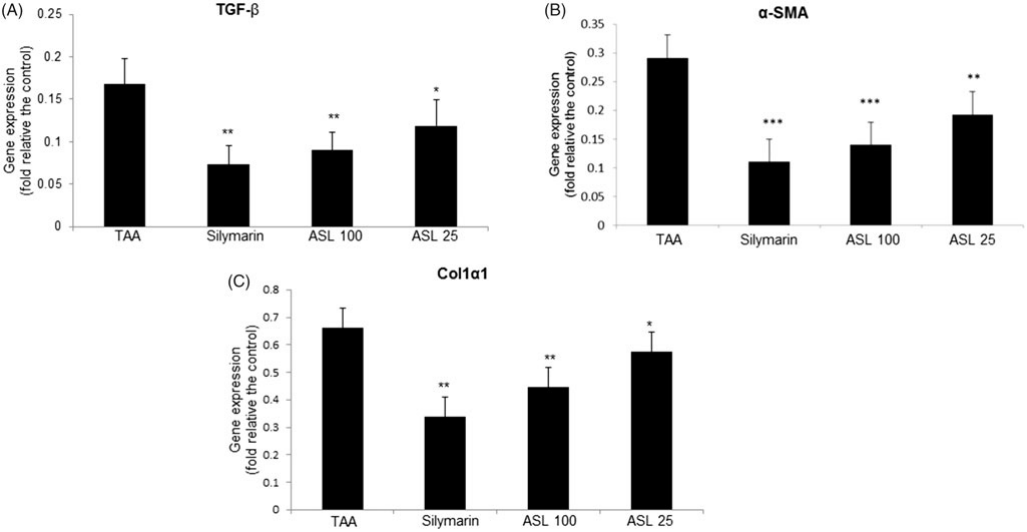
Effect of ASL on the fibrosis related gene expression in TAA-induced liver tissues. Fibrosis related gene expressions of liver tissue were determined by qualitative real-time PCR. (A) TGF-β, (B) α-SMA and (C) Col1α1. The results are expressed as normalized fold values relative to the control. Values are represented as mean ± SEM (*n* = 10) using one-way ANOVA followed by Student’s *t*-test. *p < 0.05, **p < 0.01, ***p < 0.001 as compared to TAA group. TAA: Thioacetamide.

To understand the mechanisms by which ASL presents antifibrotic effects, we further investigated the effect of ASL on the major fibrotic marker expression such as TGF-β, α-SMA and Col1α1. As shown in Figure 7, quantification of gene expression revealed an increased expression of liver fibrosis-related genes (*TGF-β*, *α-SMA* and *Col1α1*) in the TAA-induced liver tissues analyzed by *q*RT-PCR. However, the TAA-induced increase in the gene expression of TGF-β, α-SMA, and Col1α1 were suppressed significantly and approximately to half the levels by ASL at 100 mg/kg, compared with TAA alone treated group. Although ASL 100 and silymarin showed similar statistical significance in down regulating of gene expression, the values of silymarin were superior to ASL treated groups (Figure 7(A–C)).

### HPLC fingerprint analysis of ASL

**Figure 8.**
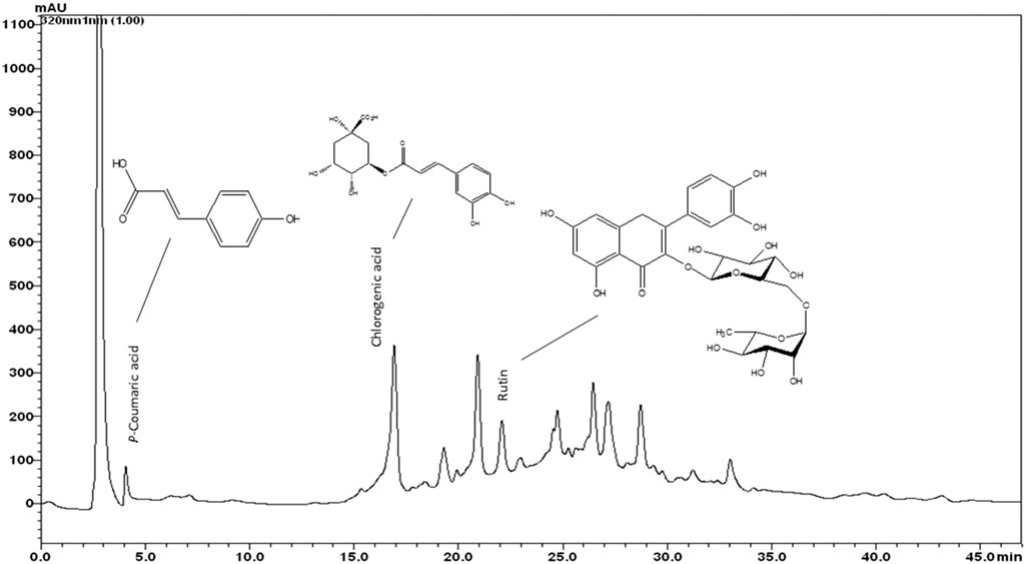
HPLC fingerprint analysis of ASL. The components of ASL were determined using an HPLC system. The reference standards of *p*-coumaric acid, chlorogenic acid and rutin were used to compare the peaks. ASL was dissolved in 80% methanol and applied to the HPLC system (Shim-pack VP-ODS column) at a flow rate of 0.15 mL/min. The analytes were detected by UV absorption at 254 nm.

The typical HPLC pattern of ASL is shown in Figure 8. The analysis revealed several distinct peaks including *p*-coumaric acid, chlorogenic acid and rutin as major peaks compared with their respective standards.

## Discussion

The liver, as the most important organ, controls various physiological processes such as metabolism, excretion, glycogen storage regulation and detoxification. Liver damage has become a serious health problem due to the utilization of several prescription drugs, exposure to various toxins including viruses, fungal products, bacterial metabolites, minerals, environmental pollutants and chemotherapeutic agents (Ha et al. 2005). Liver fibrosis results from chronic liver damage and results in excessive accumulation of ECM. The major cells in the liver, ‘HSCs’ which are involved in the production of the ECM, are activated during liver damage undergoing transdifferentiation to fibrogenic myofibroblast-like cells (Benyon and Arthur 2001). Liver cells that fall into apoptosis or necrosis after acute liver injury are replaced by newly-renewable liver cells and have a small accumulation of cell foreign substances. If the chronic liver damage is repeated, liver cell regeneration fails, and liver cells are replaced with foreign cells such as collagen. Within the liver, cells such as collagen types I, III, IV and V are likely to contain about six times more foreign fiber than normal leading to pathophysiological disturbances in liver architecture.

It is well known that abnormal activation of HSCs and dysregulation in HSC proliferation and apoptosis are key events involved in liver fibrosis (Benyon and Iredale 2000; Bataller and Brenner 2005). Therefore inhibition of HSC activation and inducing apoptosis might serve in preventing or treating liver fibrosis. In the present study, silymarin, a standardized extract of the milk thistle (*Silybum marianum*), is used as a positive control. Silymarin possesses strong antioxidant effects and has been used as a promising agent to treat alcoholic liver disease, viral hepatitis and toxin-induced liver diseases in clinical and hepatofibrotic experimental animals (Vargas-Mendoza et al. 2014). However, the complexity of the silymarin absorption, disposition, nature of various flavonoids and extensive first-pass metabolism, limitations in the silymarin treatment for severe liver damage urges researchers to discover complementary and alternative therapeutic agents. Currently, research on the development of anti-hepatofibrosis agents is primarily focused on traditional medicines especially from natural products of plant origin. In this study, *A. senescens* extract was found promising for treating hepatofibrosis in HSCs *in vitro* and TAA-induced liver fibrosis experimental rat model.

*In vitro* studies with primary HSCs revealed that ASL (0.025 and 0.1 mg/mL) inhibited the HSCs proliferation, and attenuated the associated fibrogenic events by dampening HSCs activation, and rescuing the altered morphology of primary HSCs. ASL not only inhibited activation and proliferation of HSCs but also triggered apoptosis which is potentially necessary for the prevention and treatment of hepatofibrosis (Guicciardi and Gores 2010). In an *in vivo* study, chronic TAA induction caused hepatofibrosis as observed histopathological in liver tissues. Experimentally, TAA which is readily metabolized to reactive acetamide and TAA-S-oxide react with hepatic cells causing DNA/protein damage, accumulate fatty acids and increased ROS formation (Abramovitch et al. 2011). Further liver injury was also examined by an increase in ALT and AST levels from TAA-induced liver tissue damage. AST and ALT secreted into the blood due to the instability in the liver cell membrane integrity caused by TAA-induction are the most commonly used markers of hepatocyte injury (Johnston 1999). ASL treatment (25 and 100 mg/kg) decreased the serum enzyme levels of AST and ALT that were increased by the TAA significantly (*p* < 0.05 and *p* < 0.001). It is well known that free radicals play a significant role in the development of TAA-induced liver fibrosis (Parola and Robino 2001). GSH which plays a major role as a reductant in oxidation-reduction processes is a functional biological marker in liver (Carlberg and Mannervik 1985; Fakurazi et al., 2008). In the present study, the ROS produced by the TAA, reduced the antioxidant GSH enzyme levels which was restored by ASL treatment thereby protecting hepatocytes against oxidative damage.

The ECM accumulation, particularly collagen is a major phenomenon in liver fibrosis. Hydroxyproline, a product of proline hydroxylation catalyzed by an enzyme polyhydroxylase is an amino acid unique to all the collagens and a major factor for collagen stabilization (Hanauske-Abel 2003; Krane 2008; Palfi and Perczel 2008). Therefore, the measurement of hydroxyproline content serves as an excellent standard to evaluate the extent of liver fibrosis. In the present study, TAA-induced groups showed a significant (*p* < 0.001) increase in the level of hydroxyproline and ASL at both concentrations significantly ameliorated this increase in rat liver tissues. The results suggest that ASL suppresses the TAA-induced liver fibrosis by preventing ROS generation and ECM accumulation. In addition, TAA-induced changes in liver tissue morphology were restored to normal in the ASL-treated groups, as shown by the H&E staining. Moreover, the fibrosis area was recovered in the ASL-treated groups as indicated by Picrosirius red staining and the suppression of collagen accumulation and tissue regeneration was observed.

TAA-induced liver damage mainly by the secreted reactive metabolic products of TAA not only activates HSCs but also produces fibrinogen and growth factors aiming at promoting acute liver injury to chronic hepatofibrosis (Bassiouny and Shaker 2011). Among the growth factors, TGF-β has multiple profibrogenic effects which are produced from damaged liver cells, activated Kupffer cells and platelets inducing HSCs activation and proliferation (Stalnikowitz and Weissbrod 2003). The produced TGF-β upregulates the transcription of the collagen genes (*Col1α1* and *Col1α2*) which are highly expressed in activated HSCs and observed in the damaged liver. Reports indicated that the growth factors including TGF-β1 also amplifies α-SMA expression, a specific marker for smooth muscle cell differentiation used to identify activated HSCs that show a myofibroblastic phenotype (Carpino et al. 2005; Gressner and Weiskirchen 2006; Tacke and Weiskirchen 2012). Therefore inhibition of the TGF-β1/α-SMA signaling pathway might attenuate liver fibrosis. In the present study, we evaluated the effect of ASL on the fibrotic-mediated gene expression such as TGF-β, Col1α1 and α-SMA in rat liver tissues induced by TAA. We observed that ASL (25 and 100 mg/kg) significantly suppressed the TAA-induced increase in the expression of TGF-β, Col1α1 and α-SMA in rat liver tissues. These results indicate that ASL exerts its antifibrotic action by inhibiting HSCs proliferation and activation via partly mediating through the inhibition of the TGF-β1/Smad pathway.

Traditionally, *A. senescens* is considered as an important medicinal herb used in detoxifying and restoring liver function (Kim, 1998). *Allium senescens* was also used as a healthy addition to the diet, to help in reducing blood cholesterol levels and act as a tonic to the digestive and circulatory systems. Earlier reports revealed that ASL contained several bioactive polyphenolic constituents such as chlorogenic acid, coumaric acid, ferulic acid, rutin and organosulfur garlic compounds like allicin and allinin (Parvu et al. 2010, 2011). Some of these compounds such as chlorogenic acid, rutin, coumaric acid and allicin have the ability to scavenge free radicals, regulate endogenous antioxidants status, maintain oxidative balance and possess strong hepatoprotective effects including antifibrotic effects (Rukkumani et al. 2004; Vimal and Devaki 2004; López-Revuelta et al. 2006; Magalingam et al. 2013; Ekinci Akdemir et al. 2017; Li et al. 2017). In the present study, HPLC fingerprint analysis of ASL exhibited several distinctive peaks including chlorogenic acid, *p*-coumaric acid and rutin. The various compounds identified and other active constituents present in the ASL might act individually or synergistically in delivering potent anti-hepatofibrotic effects. However, future studies on isolation and identification of each active constituent present in ASL extract and in-depth evaluation for their antifibrotic activities *in vivo* is quite essential.

In conclusion, the *in vitro* results indicated that ASL suppressed the proliferation, inhibited the ECM accumulation and triggered apoptosis in HSC-T6 cells. The morphology of HSCs was restored to their normal quiescent form. The *in vitro* results supported our *in vivo* studies where ASL administration reduced the increased serum ALT and AST biomarkers, improved the GSH content and alleviated the increased hydroxyproline levels in TAA-induced fibrotic rats. Moreover, ASL exhibited similar or superior beneficial effects in several parameters over silymarin. Our present data provided scientific evidence for the traditional claims of ASL, for its use in liver diseases. Based on these findings, we suggest that *A. senescens* might be developed as a therapeutic potential against various liver-related disorders including liver fibrosis.
